# Atomistic Modelling of Size-Dependent Mechanical Properties and Fracture of Pristine and Defective Cove-Edged Graphene Nanoribbons

**DOI:** 10.3390/nano10071422

**Published:** 2020-07-21

**Authors:** Daniela A. Damasceno, R.K.N.D. Nimal Rajapakse, Euclides Mesquita

**Affiliations:** 1 School of Engineering Science, Simon Fraser University, Burnaby, BC V5A 1S6, Canada; daniela.damasceno@usp.br; 2 Department of Materials Physics and Mechanics, Institute of Physics, University of São Paulo, Ed. Van de Graaff–ed 10-Grupo SAMPA, Rua do Matão, Travessa R, 187, São Paulo 05508-090, Brazil; 3 Department of Civil Engineering, Sri Lanka Institute of Information Technology, Malabe 10115, Sri Lanka; 4 Department of Computational Mechanics, and Center for Computational Engineering & Sciences (CCES), University of Campinas, Mendeleyev, 200-Cidade Universitária, Campinas, São Paulo 13083-860, Brazil; euclides@fem.unicamp.br

**Keywords:** cove-edges, defects, fracture, graphene, molecular dynamics, strength

## Abstract

Cove-edged graphene nanoribbons (CGNR) are a class of nanoribbons with asymmetric edges composed of alternating hexagons and have remarkable electronic properties. Although CGNRs have attractive size-dependent electronic properties their mechanical properties have not been well understood. In practical applications, the mechanical properties such as tensile strength, ductility and fracture toughness play an important role, especially during device fabrication and operation. This work aims to fill a gap in the understanding of the mechanical behaviour of CGNRs by studying the edge and size effects on the mechanical response by using molecular dynamic simulations. Pristine graphene structures are rarely found in applications. Therefore, this study also examines the effects of topological defects on the mechanical behaviour of CGNR. Ductility and fracture patterns of CGNR with divacancy and topological defects are studied. The results reveal that the CGNR become stronger and slightly more ductile as the width increases in contrast to normal zigzag GNR. Furthermore, the mechanical response of defective CGNRs show complex dependency on the defect configuration and distribution, while the direction of the fracture propagation has a complex dependency on the defect configuration and position. The results also confirm the possibility of topological design of graphene to tailor properties through the manipulation of defect types, orientation, and density and defect networks.

## 1. Introduction

**Figure 1:**
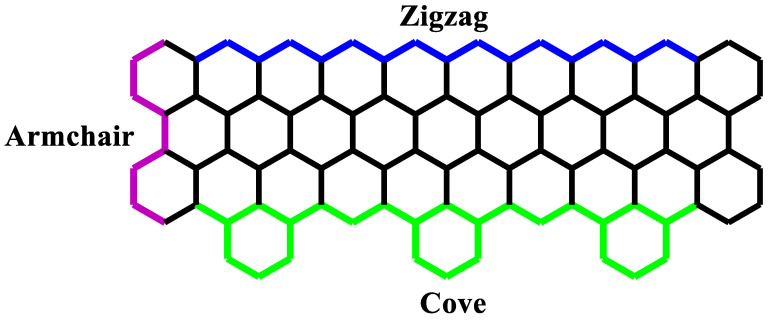
Types of edges in graphene nanoribbon.

Graphene nanoribbons [1,2] (GNR) are narrow stripes of graphene with widths normally less than 10 nm. The GNR configuration has two important structural parameters: the width and the edge patterns. The most common edges observed experimentally [2,3,4,5] are the armchair and zigzag edges, as shown in Figure 1. GNR with a controllable design of the width and edges can open bandgaps [4], which change its electronic properties [6,7] making them promising for the development of new nanoelectronic devices [8,9,10]. However, several studies [11] show that the GNR applicability is directly related to its types of edges and width. The control of these structural parameters is a challenging task during the synthesis process. To date, the bottom-up synthesis [7] of GNR is considered the most appropriate approach to produce GNR structures with controllable width and smooth edges [12,13]. This advance in the synthesis process gives GNR the potential to be used in the development of GNR-based nanoelectronics [14].

Recently, bottom-up synthesis of graphene allowed a configuration of GNR with cove-typed edges [2]. Cove-edged graphene nanoribbons (CGNRs) are a class of nanoribbons with asymmetric edges composed of a repetition of hexagons and vacancies, as shown in green in Figure 1. The cove-typed edges were observed along the zigzag GNR (ZGNR). Since their synthesis, they have been used in several interesting studies involving electronic [15,16,17] and chemical [18,19] applications.

The armchair (*ar*) and zigzag (*zz*) edges are the most investigated types by both theoretical and experimental approaches. Several studies using molecular dynamics (MD) simulations [20,21,22,23,24], atomic-scale finite element method [25] and experimental measurements [26,27] have been used to investigate the mechanical properties of GNR along the *ar* and *zz* directions. A study of the electronic properties of zigzag graphene nanoribbons with constructed edges representing pentagonal and heptagonal defects has been presented by Pincak et al. [28], whereas the effects of edge vacancies on the electronic properties of zigzag nanoribbons have been addressed by Smotlacha and Pincak [29]. Although the electronic properties of CGNR have been extensively studied [2,15], little attention has been paid to its mechanical properties, which are also very important in device design, fabrication and operation.

Defects cannot be avoided during the synthesis process of GNR; consequently, the applicability of graphene-based materials is also related to the presence of defects. In some cases, defects are introduced into the lattice intentionally, to obtain attractive properties. For example, defects such as Stone–Wales (SW) [30], 5-8-5 [31], extended line of defects (ELD) [32] and nanopores have been introduced in GNR to enhance electronic [27,33,34,35] and thermal properties [36]. On the other hand, the presence of defects reduces the mechanical strength of GNR [14,31,37], which is prejudicial for the integrity of the structures. Therefore, it is essential to study the effects of defects and edges on the mechanical properties of GNR to support the development of strong GNR-based devices.

Several studies [20,22] using MD simulations have been used to study the mechanical properties and the fracture patterns of the defective *ar* and *oz.* GNR (AGNR and ZGNR, respectively). Results [20] show that topological defects such as Stone–Wales can be introduced into the GNR without compromising the stiffness, whereas the ultimate stress is reduced. Studies [27] also show that defects make GNR more flexible. Moreover, the mechanical properties of GNR with disordered edges [38] and graphene nanowiggles [39] have been studied in details. In regarding to CGNRs, their pristine form showed to possess remarkable electronic properties just by controlling the width and the edges shape. However, studies involving their mechanical properties in the presence of topological and vacancy defects are still missing.

This paper investigates the key mechanical properties of pristine and defective CGNR. It is carried out using MD simulations based on the adaptive intermolecular reactive bond order (AIREBO) potential. Firstly, the mechanical properties of CGNR considering different sizes of width and length are presented. Second, the effects of three different edges configurations on the mechanical response of CGNR are examined. Third, the effect of the presence of topological and vacancy defects on the mechanical response of CGNR is studied. Finally, the effects of the density of divacancy and the combination of vacancy and SW1 defects on the mechanical properties of CGNR are investigated. The present study therefore provides good insight about the mechanical properties of CGNR, which are important in device design and fabrication while enabling device designers to exploit their remarkable electronic properties.

## 2. Methodology

This study is carried out through MD simulations based on the AIREBO potential [40] using the LAMMPS package [41]. AIREBO potential has been successfully used to model graphene-based nanostructures as well as predict fracture patterns [24,42]. This potential is formed by the sum of three different potentials as presented in Equation (1) [40],

(1) $$E^{AIREBO}=\frac{1}{2}\underset{a}{\sum }\underset{b\neq a}{\sum }\left[E_{ab}^{REBO}+E_{ab}^{LJ}+\underset{c\neq a,b}{\sum }\underset{d\neq a,b,c}{\sum }E_{cabd}^{tors}\right]$$

where the indices *a*, *b*, *c* and *d* refer to individual atoms; $E_{ab}^{REBO}$ is the reactive empirical bond order (REBO) potential [43], which represents the energy stored in the bond between the atoms *a* and *b*; $E_{ab}^{LJ}$ is the Lennard-Jones potential, which considers the non-bonded interactions between atoms and $E_{cabd}^{tors}$ includes the energy from torsional interactions between atoms. In addition, $E^{AIREBO}$ requires a value for the cut-off radii, which is set to 2.0 Å based on the previous studies [24,42].

**Figure 2:**
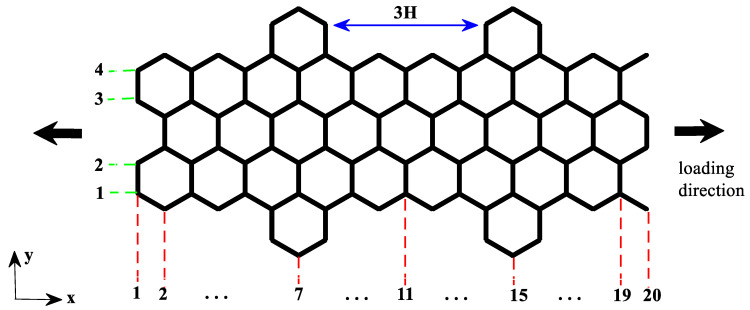
Schematic of cove-edged graphene nanoribbons (CGNR) with the definition of parameters NL (number of carbon–carbon dimer lines along the ribbon length (x direction)), NW (number of carbon–carbon dimer lines along the ribbon width (y direction)) and *n*H (distance between the hexagons at cove-typed edges).

Figure 2 shows a schematic of cove-edged graphene nanoribbon. The parameter NW corresponds to the number of carbon–carbon dimer lines along the ribbon width (y direction), while the parameter NL corresponds to the number of carbon–carbon dimer lines along the ribbon length (x direction). This convention is in concordance with previous studies [18,44,45]. The NW and NL parameters define the CGNR size. The example in Figure 2 corresponds to NW = 4 and NL = 20. The parameter *n*H corresponds to the distance between the hexagons at cove-typed edges. For example, 3H means that three hexagons are missing; this parameter is used to represent the cove configuration of the edges. To study the mechanical response of a CGNR, the periodic boundary conditions are considered in the x-direction. Uniaxial tension tests are simulated along the zigzag direction by applying strain at a rate of 0.001 ps^−1^ in the x-direction. All molecular dynamics (MD) simulations are carried out at temperature of 1 K with temperature controlled by the Nose-Hoover thermostat. This thermostat has been widely used for carbon systems and is known to yield stable and efficient computations [20,21,22,24,37,40,44]. The timestep was 0.5 fs and a uniaxial tension was applied after the system was relaxed at zero pressure through isothermal–isobaric ensemble (NPT).

The results are presented in the form of stress–strain diagrams. The strain measure is defined by the displacement, $\Delta L_{x}$, divided by the original mesh length $L_{x}^{0}$:

(2) $$\varepsilon_{x}=\frac{\Delta L_{x}}{L_{x}^{0}}$$

where *x* represents the Cartesian coordinate axis, $\Delta L_{x}=L_{x}-L_{x}^{0}$, and $L_{x}$ is the actual length of the CGNR.

The stress values are obtained by averaging virial stress, $\sigma_{xy}$ of each carbon atom, expressed by [46]

(3) $$\sigma_{xy}=\frac{1}{V}\underset{\alpha }{\sum }\left[\frac{1}{2}\overset{N}{\underset{\beta =1}{\sum }}\left(R_{x}^{\beta }-R_{x}^{\alpha }\right)F_{y}^{\alpha \beta }-m^{\alpha }v_{x}^{\alpha }v_{y}^{\alpha }\right]$$

where the indices *x* and *y* denote the Cartesian coordinate axes; *V* is the volume used for the stress calculation considering the thickness as 3.4 Å; $R_{x}^{\beta }$ is the location of atom β along the *x* axis; $R_{x}^{\alpha }$ is the location of atom α along the *x* axis; $F_{y}^{\alpha \beta }$ is the force acting on atom α due to its interaction with the neighbours β in the *y* direction; $m^{\alpha }$ is the mass of atom $\alpha \mathrm{and}\;v_{x}^{\alpha }$ and $v_{y}^{\alpha }$ are the velocities of atom α along the *x* and *y* directions, respectively.

## 3. Results and Discussion

### 3.1. Validation of MD Simulation

**Figure 3:**
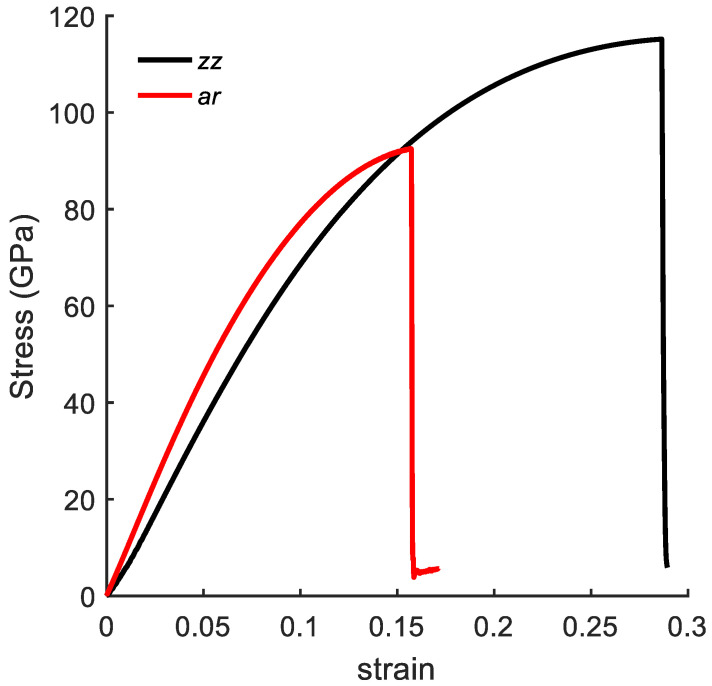
Stress–strain curves of pristine graphene sheet under uniaxial tension along *ar* and *zz* directions.

To validate the numerical model, the stress–strain curves of a pristine bulk graphene sheet under uniaxial tension are compared with the values presented in the literature. A pristine graphene sheet with dimensions of 48.9 Å × 48.4 Å was considered. Figure 3 shows the stress–strain curves along the *ar* and *zz* directions. The ultimate tensile strength obtained from MD simulations was 92 GPa and 115 GPa and the fracture strain was 0.16 and 0.28 along the *ar* and *zz* directions, respectively. The MD results along the *zz* direction agreed well with the experimental [47] results for the fracture stress and fracture strain, which were 130 ± 10 and 0.25, respectively. Moreover, these results were also in agreement with the MD simulation results presented in the literature, ranging from 83 to 137 GPa and 0.12 to 0.27 along the *ar* direction, and 98 to 138 GPa and 0.12 to 0.28 along the *zz* direction [48,49].

### 3.2. Size Effects on Mechanical Response of CGNR

In this section, we focused on the mechanical response of CGNR under uniaxial tension loading considering four different values NL and NW. The CGNR samples have the distance between the hexagons equal to 2H.

**Figure 4:**
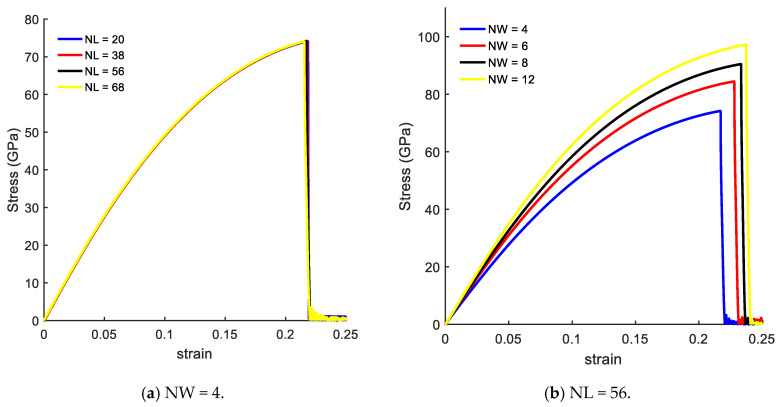
Size effects on the mechanical response of CGNR (2H).

Figure 4 presents the size effects on the mechanical response of CGNR in a form of stress–strain curves. Figure 4a shows the stress–strain curves with fixed width of NW = 4, and length varying from NL = 20–68. The mechanical response of CGNR shows hardly any dependency on the length as the periodic boundary conditions were used in the length direction. Such behaviour also confirms the validity and correct physical response of the MD model of CGNR. Moreover, this behaviour was similar to the response previously observed for AGNR and ZGNR [21].

Figure 4b shows the stress–strain curves with a fixed length of NL = 56, and width (NW) varying from 4 to 12. Here, the mechanical response of CGNR shows interesting behaviour with changing widths; CGNR became stronger and more ductile as the width increased. It is due to the edge effects that become significant with decreasing NW and bond breaking becomes easier along the width direction after the first bond breaks. It should be noted that strength and fracture strain of nanoribbons shown in Figure 4 were smaller than the ultimate strength and fracture strain of a pristine sheet shown in Figure 3. However, as NW increased the strain-curve tended to approach the response of a pristine sheet. Comparing pristine zigzag graphene sheet and CGNR with NW = 12 the ultimate tensile strength was 115 GPa and 97 GPa and the failure strain was 0.28 and 0.24, respectively.

Moreover, the mechanical response of CGNR with varying widths was different from the response previously observed for AGNR and ZGNR. Chu et al. [21] and Damasceno et al. [25] concluded that AGNR shows little size dependency with width whereas the size dependency with width is higher in the case of ZGNR. However, both authors [21,25] showed that in the case of the ZGNR as the width increased the ultimate strength and the failure strain decreased. Moreover, other studies also concluded that the ultimate strength is larger as the width is decreased [38,39], while the fracture strain is increased. Therefore, we can conclude that ZGNR with cove-typed edges becomes stronger as the width increases, as well as increase its fracture strain in contrast to the behaviour presented by the ZGNR in the literature. This behaviour is relevant to the design of CGNR devices as the lower mechanical strength and ductility could have a direct effect on the device operation and reliability.

### 3.3. Edge Effects on the Mechanical Response of CGNR

**Figure 5:**
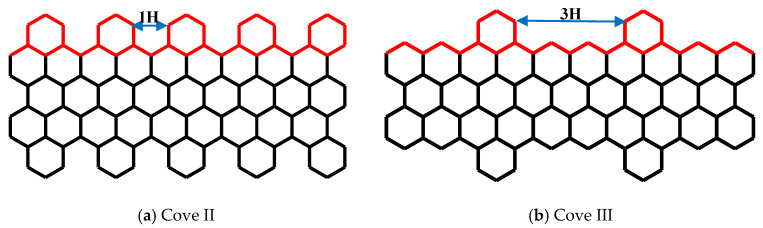
Schematic of CGNR corresponding to (**a**) 1H and (**b**) 3H.

The results presented in Figure 4 show that the edges played a different role on the mechanical response of CGNR when compared to the effect of edges on the response of AGNR and ZGNR. In this regard, it is important to examine how the behaviour observed in Figure 4 depended on the cove-edge configuration shown in Figure 2 (i.e., the distance between the hexagons). Therefore, in the next example, we investigated the influence of parameter *n*H by comparing the results for 1H and 3H. For each parameter *n*H, the CGNR length was fixed with NL = 56 and the width (NW) varies from 4 to 8. These two configurations were called Cove II and Cove III as shown in Figure 5a,b, respectively. In addition, we investigated the influence of higher values of the parameter *n*H by considering CGNRs with NL = 56 and NW = 4 and NW = 8.

**Figure 6:**
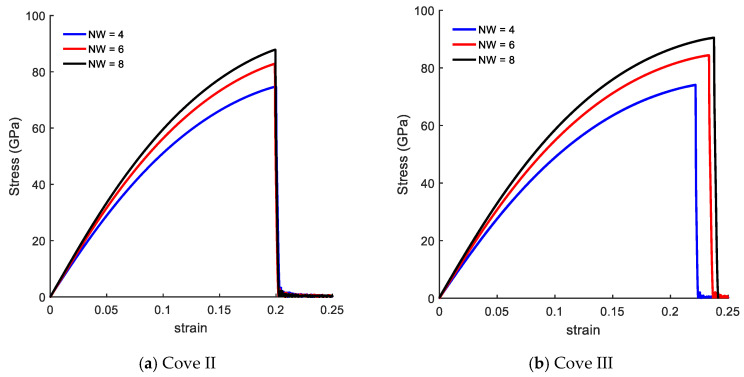

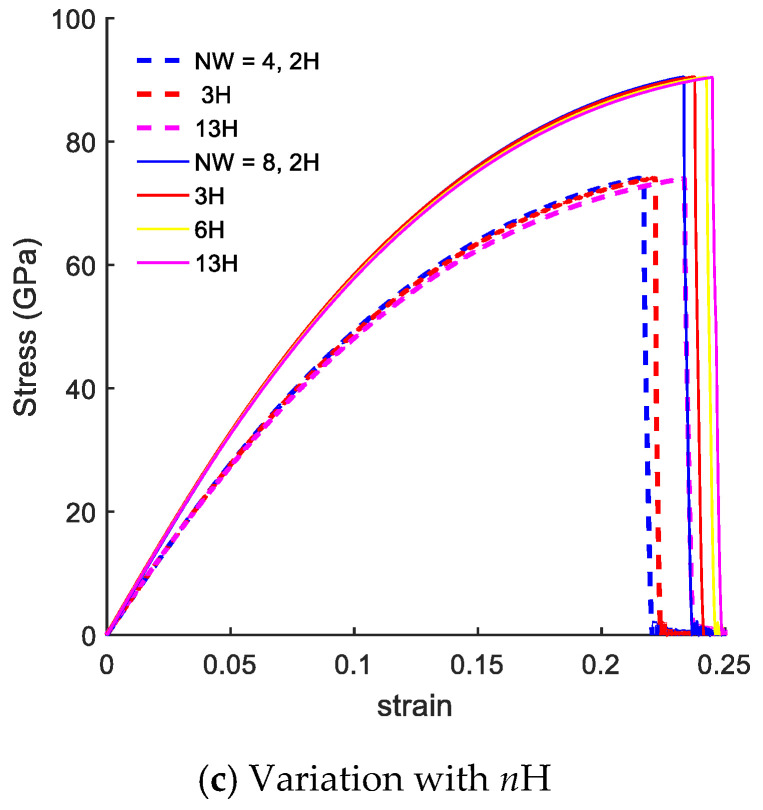
Stress–strain curves of (**a**) Cove II, (**b**) Cove III and (**c**) for different *n*H values.

The mechanical response of the Cove II and Cove III models is presented in Figure 6. Comparing to Figure 4b, the stress–strain curves shown in Figure 6 also demonstrated that as the width increased the CGNR became stiffer irrespective of the *n*H value but no change in ductility was observed for the lowest value of *n*H (i.e., Cove II). In the case of Cove III, the stress–strain curves shown in Figure 6b were very close to the curves shown in Figure 4b indicating that the spacing between the hexagons in the cove-edge was not a significant factor in the mechanical response. It is also noted that strength values in Figure 6a were also not substantially different (10%) from the strength values corresponding to 2H and 3H configurations. Moreover, the same behaviour was observed for higher values of *n*H, as presented in Figure 6c, where dashed lines corresponded to the stress–strain curves of CGNR with width NW = 4 and solid lines with width NW = 8. In both cases, the ultimate fracture stress remained the same for different *n*H, while the failure strain was slightly increased with increasing *n*. It could be concluded that, for a given width NW, CGNR presents an almost constant characteristic mechanical behaviour when the distance between the hexagons at the edge were at least spaced by three hexagons, i.e., *n* ≥ 3.

**Figure 7:**
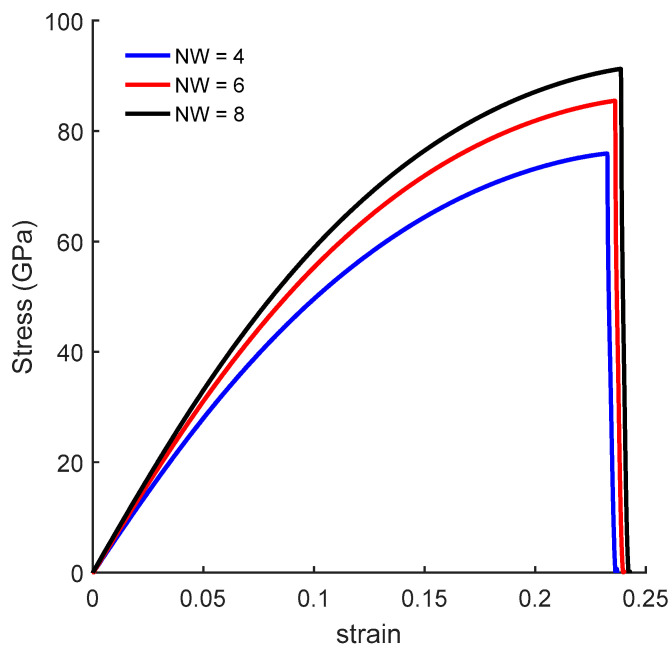
Stress–strain curves of CGNR with mixed edges.

To further investigate the effect of the cove-edge geometry, the next example considers a mixed cove-edge configuration with one side identical to the Cove II edge geometry while the opposite edge is represented by the Cove III geometry. The mechanical response of this CGNR is shown in Figure 7. The stress–strain curves shown in Figure 7 show similar behaviour to the curves shown in Figure 4b and Figure 6b; as the width increased the CGNR became stiffer but the variation of ductility with width was negligible. An important observation from Figure 4, Figure 6 and Figure 7 is that the mechanical strength of CGNR showed a minor dependence on the cove-edge configuration with strength and ductility showing less than 15% variation.

### 3.4. Topological and Vacancy Defects in CGNR

**Figure 8:**
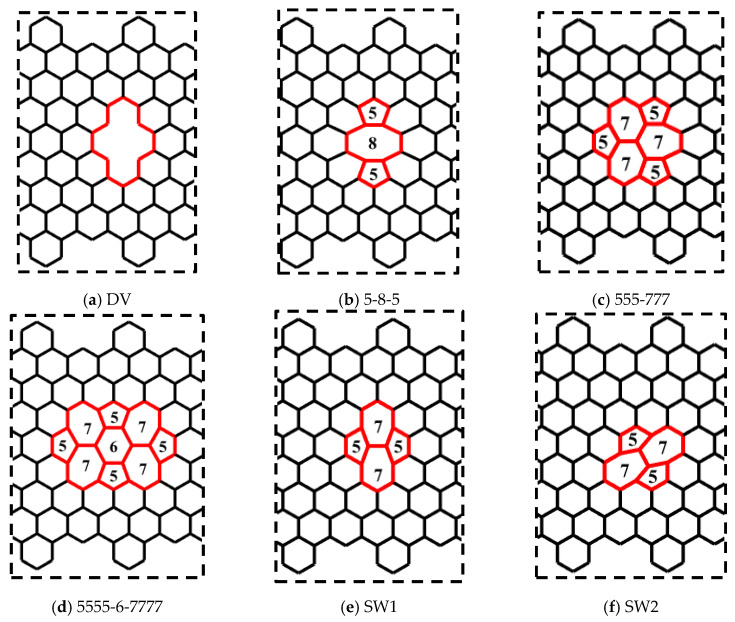
Topological and vacancy defects embedded in CGNR.

In this section, the effects of the topological and vacancy defects on the mechanical behaviour of CGNR were presented. The topological defects break the symmetry of the hexagonal lattice, while the sp^2^ covalent bonds were maintained. The vacancy defects were obtained by removing atoms from the lattice and as a result the sp^2^ covalent bonds around the defects were not maintained. The topological and vacancy defects considered in this study are shown in Figure 8. Figure 8a shows a divacancy (DV) defect obtained by removing two atoms from the center of the lattice. Figure 8b–d shows topological defects known as 5-8-5, 555-777 and 5555-6-7777. Figure 8e,f shows the Stone–Wales defects commonly identified as SW1 and SW2. To study the effect of defects on the mechanical behaviour of CGNR, each defect shown in Figure 8 was placed at the centre of a CGNR with geometry defined by NL = 56 and NW = 8. In all MD simulations, the cove-type edges had hexagons spaced at 2H, and the length of a standard carbon–carbon bond was considered initially as 1.396 Å. Note that initial bond lengths were different for certain bonds and rotated carbon–carbon bonds associated with topological defects.

**Figure 9:**
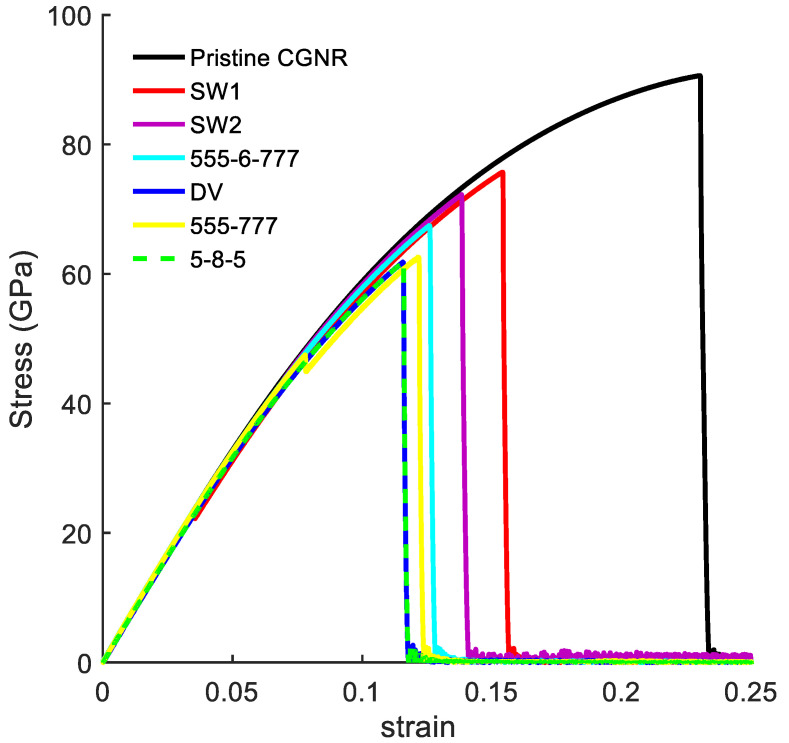
Stress–strain curves for the defects: DV, 5-8-5, 555-777, 5555-6-7777, SW1, and SW2.defects.

Figure 9 shows the mechanical response of CGNR under uniaxial tension loading. The stiffness (elastic modulus) of CGNR shows minor influence of defects and gradually became nonlinear as the strain increased beyond 5%. However, all types of defects had a significant effect on the ultimate strength and ductility of CGNR when compared to a pristine CGNR of identical size. For a pristine CGNR, the ultimate tensile strength was 90.6 GPa and the fracture strain was 0.23. Reductions in strength and fracture strain were highest for the 5-8-5, DV and 555-777 defects followed by the 5555-6-7777, SW2 and SW1. The SW1 configuration was mechanically stronger compared to all other defects. Here, it is interesting to note that the same behaviour was observed for the zigzag graphene with the SW1 defect [50]. Compared to a pristine CGNR, the strength was reduced from 90.6 to 75.7 GPa and the failure strain from 0.23 to 0.15 for a SW1 defect, whereas for a 5-8-5 defect the strength reduced to 61.8 GPa and the strain to 0.12. For a better understanding of the behaviour presented in Figure 9, it is useful to discuss the fracture patterns observed in the Visual Molecular Dynamics package [51] during the MD simulations. Firstly, we discussed the fracture patterns of the defects with higher ultimate stress, which correspond to the defects SW1, SW2 and 5555-6-7777.

**Figure 10:**
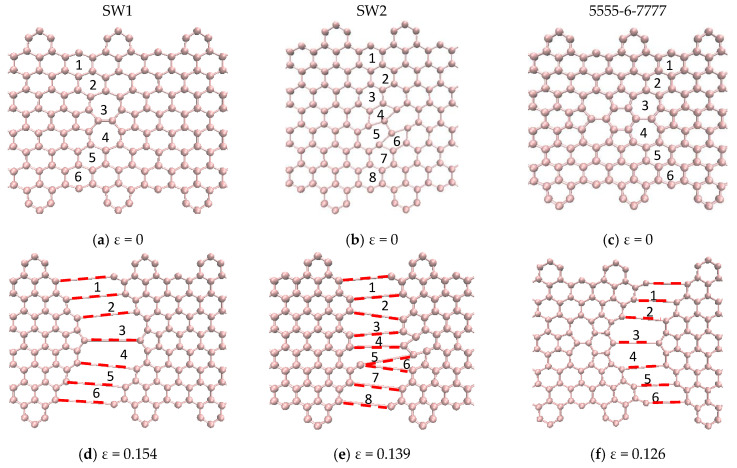
Failure patterns and related atoms and bonds of SW1, SW2 and 555-6-777 defects.

Figure 10 shows a pictorial representation of the atomic structure of the CGNR with selected defects obtained from the Visual Molecular Dynamics (VMD) package [51] just prior to the failure point shown in Figure 9. It should be noted that this figure does not show the actual bond lengths but only a visualization of the atoms and bonds that are involved in the failure. Therefore, the red broken lines illustrate the broken bonds of the system, but they do not represent the actual bond lengths. Such representations are commonly used in molecular simulations to illustrate failure patterns, etc., e.g., [22,46]. It is observed that the defects SW1 (Figure 10a) and 5555-6-7777 (Figure 10c) have a horizontal plane of symmetry and two pentagons at the front of the defects. In both cases, only the bonds of four hexagons and two heptagons get stretched and eventually break. However, for a SW1 defect the fracture runs through the two heptagons sparing the pentagons at the fronts as shown in Figure 10d and the bonds shared by the heptagon–heptagon and the heptagon–hexagon were the first to break. For a 5555-6-7777 defect, the fracture does not cut across the defect but involve only the two heptagons on the right side of the defect and the hexagons above and below them as shown in Figure 10f. Here again, the fracture initiates at the bonds shared by heptagon–heptagon and heptagon–hexagon cells. It is interesting to note that the bonds of the central hexagon and none of the pentagons were broken at the failure point. The SW2 defect is like a SW1 defect in terms of the atomic structure of the defective cells but it does not have a horizontal plane of symmetry. As a result, the fracture cuts through five hexagons, two pentagons and one heptagon. The fracture starts on the bonds shared by the pentagon–heptagon and pentagon–hexagon cells. It seems out of the three defects shown in Figure 10, SW1 has the minimum number of defective cells and together with its symmetry make it the strongest and most stretchable configuration followed by SW2 and 5555-6-7777, which has a higher number of defective cells.

**Figure 11:**
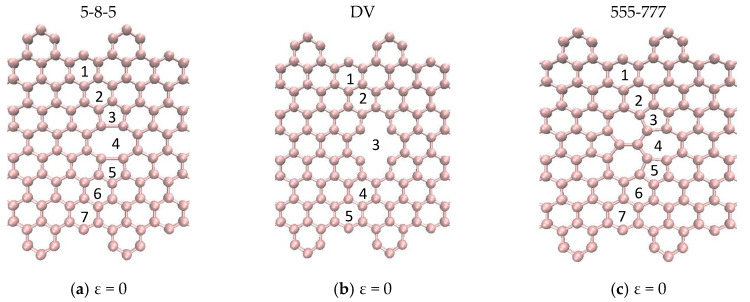

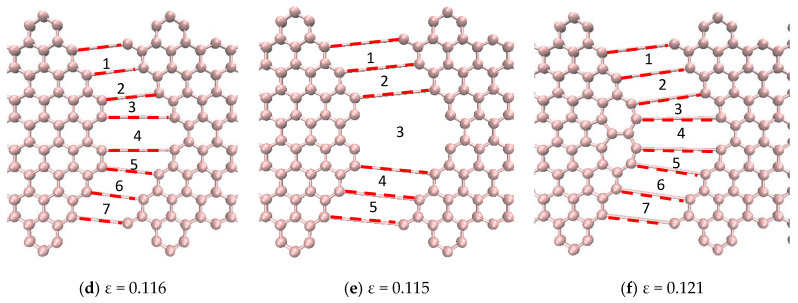
Failure patterns and related atoms and bonds of 5-8-5, DV and 555-777 defects.

The defects shown in Figure 11a–c had similar fracture propagation as in Figure 10. Here again, the red broken lines illustrate the broken bonds of the system, but they do not represent the actual bond lengths. Fracture propagates through both sides turning to the left side reaching the edges in the interval between of the hexagons. For the 5-8-5 defect, the fracture started on the bonds shared by the pentagon–octagon and the pentagon–hexagon and these bonds aligned with the loading direction relatively easily. In the case of the DV defect, the fracture started on the bonds shared by the vacancy and the hexagons. It obviously had the least resistance to failure as only six bonds were involved, and they aligned easily with the loading direction. The fracture in the 555-777 defect started on the bonds shared by the pentagon–heptagon and pentagon–hexagon. It is interesting to note that in the case of the 555-777 defect, the fracture could start on the bonds shared by the heptagon–heptagon and heptagon–hexagon as in the case of SW1 and 5555-6-7777 defects. However, due to the atomic configuration of the 555-777 defect the fracture started at different bonds. However, its strength and fracture strains were quite close to the values of 5555-6-7777 defect and slightly higher than the DV and 5-8-5 defects.

Our results revealed that the mechanical response of defective CGNR had a complex dependency on the defect configuration. Highest fracture stress and failure strain were observed for the cases where the fracture started on the bonds shared by heptagon–heptagon and heptagon–hexagon and the number of defective cells was fewer as in the case of SW1, SW2 and 5555-6-7777. Lower fracture stress and failure strain were observed for the cases where the bonds easily aligned parallel to the loading direction during the initial stages of stretching such as in vacancies and pentagon–octagon bonds. Moreover, studies have shown that the bond breaking mechanism is influenced by the amount of covalent bond loading distribution and the corresponding stretching that occurs due to the atom lattice deformation pattern. A discussion on this aspect can be found in Babicheva et al. [52].

### 3.5. Vacancy Effects on the Mechanical Response of CGNR

**Figure 12:**
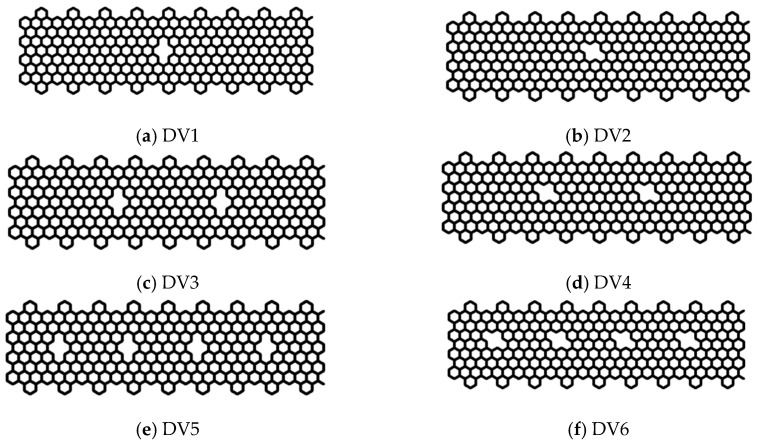
Geometry of CGNR with single and multiple DV defects.

One of the most common defects in the GNR [53] is caused by atomic vacancies. Several studies [22,36,54] have examined the effects of vacancies, such as divacancy (DV) and other vacancy configurations, on the mechanical and electronic properties of GNR along the *ar* and *zz* directions. In this section, we extended the single DV defect study presented in the preceding section to examine the mechanical response of a defective CGNR containing a single DV defect with different orientations and multiple DV defects. In addition, we examined the possibility of topological design of CGNR for strength enhancement by combining the DV and SW1 defects, as well as the manipulation of the fracture pattern using these defects. Figure 12 shows six configuration of CGNR with single and multiple DV defects. These configurations are labelled as DV1, DV2, DV3, DV4, DV5 and DV6.

**Figure 13:**
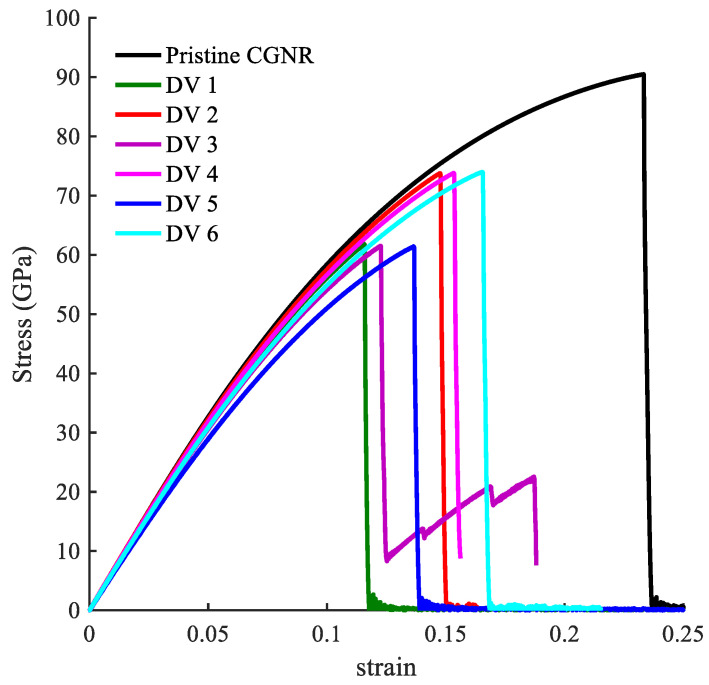
Stress–strain curves of CGNR with single and multiple defects.

**Figure 14:**
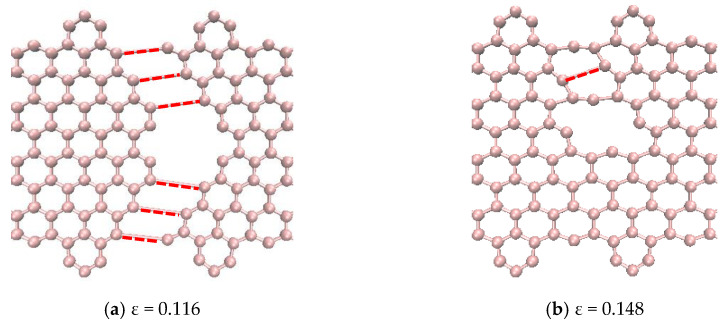
Failure patterns and related atoms and bonds of DV1 and DV2 defects.

The stress–strain curves of CGNR with single and multiple DV defects, shown in Figure 12, are depicted in Figure 13. Figure 14 compares the fracture patterns of DV 1 and DV 2 defects. It is observed that CGNR with inclined DV defects (DV2, DV4 and DV6) were stronger and more ductile compared to the ribbons with vertical DV defects (DV1, DV3 and DV5). It is also interesting to note that DV 1 had the lowest strength and fracture strain (61.8 GPa and 0.116) followed by DV3 and DV5. In general, the addition of more defects tended to increase the strength slightly and results in a noticeable improvement in ductility. For example, DV6 had the highest strength and ductility (73.9 GPa and 0.165) compared to other five configurations. This observation supported the emerging idea of topological design of 2D materials by manipulating defects to tailor properties.

**Figure 15:**
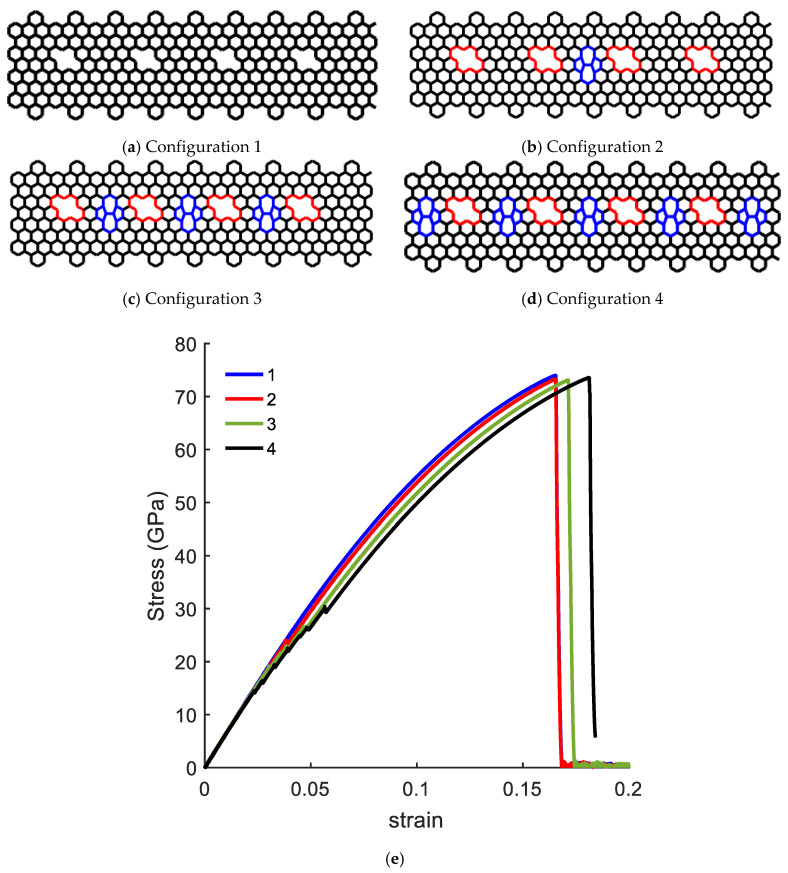
Configuration of CGNR (**a**–**d**) with defect combinations and (**e**) corresponding stress–strain curves.

In the next example, the influence of a network of simple defects was examined to further explore the emerging idea of topological design of 2D materials. We considered a CGNR with a set of inclined DV defects (DV6 configuration in Figure 12 and added SW1 defects to examine the response of the resulting CGNR. Four configurations of CGNR were considered as shown in Figure 15a–d. The mechanical response of these configurations was presented in Figure 15e. The results revealed an interesting behaviour with the combination of the defects. The ultimate strength practically remained the same as the number of SW1 increased, whereas the fracture strain had a noticeable increase. Therefore, it is concluded that the combination of inclined DV and SW1 defects could increase the mechanical flexibility without compromising the stiffness of CGNR.

## 4. Conclusions

We investigated the effects of edges, size and defects on the mechanical response of CGNRs by using molecular dynamics simulations. The results revealed that the CGNRs were generally mechanically weaker and less ductile than pristine zigzag graphene. The variation of strength and ductility with ribbon width of CGNR was qualitatively different from a ZGNR. The cove-edge geometry characterized by the distance between the adjacent hexagons shows a relatively minor influence (<15%) on strength and ductility. The mechanical response of defective CGNRs shows complex dependency on the defect configuration, while the fracture pattern was strongly influenced by the defect configuration, position and orientation. The SW1 and SW2 defects were superior to other traditional defects and the DV defects had the lowest strength and fracture strain. It is shown that a change in the orientation of a DV defect could result in a substantial increase in the strength and fracture strain. The presence of multiple DV defects can improve both strength and fracture strain while addition of SW1 defects in between DV defects can improve ductility without compromising strength. The results obtained in this study support the idea of topological design of 2D materials using defects to tailor mechanical properties.
